# Infoveillance and bibliometric analysis of COVID‐19 in Nigeria

**DOI:** 10.1002/puh2.77

**Published:** 2023-03-21

**Authors:** Jimoh Amzat, Kehinde Kazeem Kanmodi, Eyinade Adeduntan Egbedina

**Affiliations:** ^1^ Department of Sociology Usmanu Danfodiyo University Sokoto Nigeria; ^2^ Department of Sociology University of Johannesburg Johannesburg South Africa; ^3^ School of Health and Life Sciences Teesside University Middlesbrough UK; ^4^ Cephas Health Research Initiative Inc Ibadan Nigeria; ^5^ Medical Research Unit Adonai Hospital Karu Nigeria; ^6^ Faculty of Dentistry University of Puthisastra Phnom Penh Cambodia

**Keywords:** bibliometric analysis, coronavirus, Google search, infoveillance study, research, information search, COVID‐19, pandemic, Nigeria

## Abstract

**Background:**

Infectious diseases often come with enormous fear because of their ability to spark and spread. The same for COVID‐19, which WHO declared a pandemic in February 2020 after a record spread in multiple countries. The global world of information and social media plays a major role in the pandemic. Hence, this study aims to analyse the patterns of internet search and research interests on COVID‐19 in Nigeria.

**Methods:**

This is an infoveillance and bibliometric research about COVID‐19 in Nigeria using systemic search through Google Trends to obtain COVID‐19 information prevalence and research incidence through bibliometric analysis using SCOPUS database. The data obtained were analysed using the Microsoft Excel 2021 software. Descriptive statistics (frequencies, mean, range and mode) were used for the summarisation of the data. The findings were presented using texts, tables, charts and maps.

**Results:**

The information search spike started 1 week before the first index case. Search volume index inequalities were observed across the country, with the northern Nigeria having a higher search volume for COVID‐19. This study also uncovered several top search terms, including “COVID‐19,” “COVID loan” and “vaccine,” and queries, including “COVID‐19 Nigeria,” “COVID loan” and “COVID‐19 in Nigeria,” among others, which showed critical infodemiologic concerns in Nigeria. The interests of Nigerian researchers concerning COVID‐19 cut across various disciplines. The top three subject areas with the most significant volume of these publications were Medicine, Social Sciences and Biochemistry. This study found extensive research collaboration with over 150 countries coupled with external funding.

**Conclusion:**

As internet search spikes reflect population health concerns and information‐wish, understanding the infodemic patterns and search terms will influence mass media regulators and health authorities to be vigilant and tackle the spread of misinformation. Nigeria's research resilience depicts great potential, hence, a call for improved local funding for research and development.

## INTRODUCTION

The coronavirus disease of 2019 (COVID‐19) remains a global health shocker with enormous socioeconomic devastations. It was a major health emergency that shocked the world, like the Black Death of 1348‐135 and influenza (1918 and 1920), each killing over 50 million people [1, 2]. The modern era has faced a tremendous threat of infectious diseases, of which the human immune‐deficiency virus, which started in the early 1980s, has been the most horrific. So far, it has infected more than 70 million people and killed more than half of them [3]. The previous international health emergencies of the last decade (2012–2022) include the Avian flu, Zika virus (2015–2016) and Ebola crisis in West Africa (2014–2015) – the diseases were mostly zoonoses, which created panic waves at different times across the globe [4]. Infectious diseases often come with enormous fear because of their ability to spark and spread through physical contact with infected persons and their body fluids. The same for COVID‐19, which WHO declared a pandemic in February 2020 after a record spread in multiple countries. As of 7 July 2022, COVID‐19 has been reported in almost all countries, infecting over 557 million people and killing more than 6.3 million people [5]. One significant dimension of the pandemic is the infodemic.

The global world of information and social media plays a major role in the pandemic. Infodemic refers to the availability of a flood of information, either credible or not, which influence public perception of a trending issue. Efforts are geared towards making accurate COVID‐19 information available through infoveillance. The rise of search engines and social media (including Twitter, Facebook, WhatsApp, TikTok, YouTube, Instagram, Snapchat, LinkedIn and among others) has made information widely available, sometimes beyond human imagination. No wonder the first significant way of sustaining public interest is to make information available that could be assessed through various social media platforms. In the COVID‐19 era, people's responsiveness must be sustained by seeking more information about the various aspects of the pandemic. This is why there is the development of Infodemiology as the “science or study of the determinants of health information and misinformation” [6] and their implications for population health interventions.

Nigeria is the primary focus of this research. The country recorded its first case of COVID‐19 on 27 February 2020. Since then, Nigeria has recorded over 257,000 COVID‐19 cases, with over 3000 deaths. Before the first reported case, panic waves and curiosities were disseminated through the media. The media are embedded with values and issues [7] and sometimes with media hype and uncertainties, which necessitate seeking more information, primarily through social media and search engines. The scientific community is also motivated to research emerging issues for more clarification and understanding. It was noted that it is critical to analyse “how people search and navigate the Internet for health‐related information, as well as how they communicate and share this information [8]” to get crucial insights into health‐related (online) behaviour of populations. Although online information is virtual, it invariably influences offline behaviours. Health‐related lay perception and anxieties can be obtained through infoveillance. Therefore, this is an infoveillance and bibliometric research about COVID‐19 in Nigeria using systemic search (via Google Trends) analysis to obtain COVID‐19 information prevalence, spike dynamics and research incidence to document (via bibliometric analysis) the trending nuances and research outputs.

## METHODS

### Study design

This study adopted the use of infoveillance and bibliometric review approaches. These two approaches have been used extensively in the literature [9, 10, 11, 12].

### Databases

The infoveillance approach used was Google Trends database, whereas the SCOPUS database was used for the bibliometric analysis. These two databases have been extensively used in the literature [10, 11, 12, 13, 14, 15].

Asides Google Trends search database, there are other online search databases that can be used for infoveillance studies; however, Google Trends is the most preferred database because it is the oldest one and it has broader global coverage, usage and comprehensiveness compared to other databases [10, 11]. Additionally, the analysis of infoveillance data obtained from Google Trends provides insightful information on public interests; it also explores the epidemiological features and dynamic variations in infectious diseases such as COVID‐19 [10, 14, 15]. Hence, the above justifies the use of Google Trends for this study.

The SCOPUS research database was solely used for the bibliometric analysis part of this study because the database is the largest, oldest and most comprehensive research database in the world [12, 13]. Another justification for the sole use of SCOPUS was that the utilisation of multiple databases often limits the opportunity to perform a rich bibliometric analysis due to the difficulty in synchronising bibliometric data obtained from multiple databases [13].

### Data search and extraction

On 04 July 2022, we searched Google Trends using the search term “COVID‐19.” In the search process, the search command was set so that it will scoop out data on COVID‐19 searches in Nigeria alone, from 2004 to 26 June 2022, all categories of search, and web searches [11]. From the search, infoveillance data such as search volume index (SVI), names of political states, rising queries, top queries, rising topics, top topics and time (months and years) were obtained concerning COVID‐19. The data obtained were exported in a comma‐separate value (.csv) format for analysis.

On the same day – 04 July 2022 – we searched SCOPUS, with the aid of truncations, using this search string: “(TITLE‐ABS‐KEY (covid*) OR TITLE‐ABS‐KEY (sars‐cov‐2) OR TITLE‐ABS‐KEY (coronavir*) OR TITLE‐ABS‐KEY (corona AND vir*) AND AFFILCOUNTRY (Nigeria).” The following bibliometric data were obtained from the search: citation data, bibliographical data, funder details, *h*‐index and journal CiteScore 2021). The citation data included authors’ names, journal names, publication type, citation count and volume, year, issue and page numbers of those publications. The bibliographical data included institutional and country affiliations, publication language and subject areas. The CiteScore 2021 is a ranking of the impact of a journal, and it is determined by SCOPUS using this formula:

$$\mathrm{CiteScore}\;2021=\frac{\begin{array}{c} \mathrm{number}\;\mathrm{of}\;\mathrm{citations}\;\mathrm{received}\;\mathrm{in}\;\text{2018--2021}\;\mathrm{to}\;5\\ \mathrm{published}\;\mathrm{document}\;\mathrm{types}\;\left(\mathrm{articles},\;\mathrm{etc}.\right)\;\mathrm{by}\;a\;\mathrm{journal}\;\mathrm{in}\;\mathrm{the}\\ \mathrm{same}\;4\;\mathrm{years} \end{array}}{\begin{array}{c} \mathrm{total}\;\mathrm{number}\;\mathrm{of}\;\mathrm{documents}\;\mathrm{indexed}\;\mathrm{in}\;\mathrm{SCOPUS}\;\mathrm{and}\\ \mathrm{published}\;\mathrm{in}\;\text{2018--2021} \end{array}}$$

Lastly, the *h*‐index is a measure of influence level, and it is defined as the total number of *h* publications cited at least *h* times [9].

### Data analysis

The data obtained were analysed using the Microsoft Excel 2021 software. Descriptive statistics (frequencies, mean, range and mode) were used for the summarisation of the data. The findings were presented using texts, tables, charts and map.

### Ethical considerations

This study did not collect data from animals or humans; all data collected were from open repositories which do not require prior permission before access. Hence, approval from an ethics committee to conduct this study is not applicable.

## RESULTS

### Search interest

**FIGURE 1:**
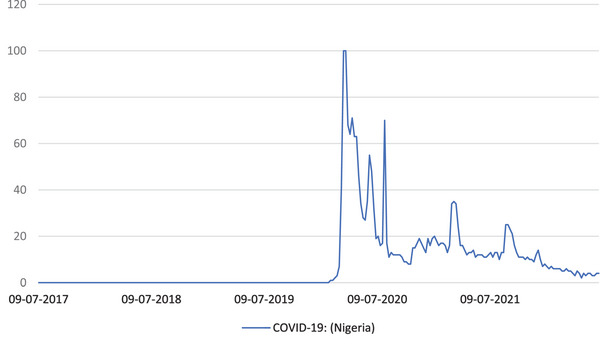
The trend of search volume indexes (SVIs) of COVID‐19, in Nigeria, on Google Trends.

Figure 1 shows the trend of SVIs of COVID‐19, in Nigeria, on Google Trends from 09 August 2017 to 26 June 2022. From 09 August 2017 to 02 February 2020, the SVI of COVID‐19 was zero. At periods latter to 02 February 2020, the SVIs were >1. There were multiple spike periods on COVID‐19 search in the year 2020–2022; however, the period with the greatest spike was 22 February 2020.

All the states in Nigeria had a COVID‐19 SVI of at least 10 over the analysed period. The state with the lowest SVI was Lagos, whereas that with the highest SVI was Zamfara (Table 1).

**TABLE 1:** Ranking of states in Nigeria based on search volume index (SVI).

Rank	State	SVI	Rank	State	SVI	Rank	State	SVI	Rank	State	SVI
1st	Zamfara	100	11th	Benue	44	21st	Cross River	23	31st	Rivers	16
2nd	Yobe	93	12th	Adamawa	37	21st	Kaduna	23	31st	Abia	16
3rd	Kebbi	85	13th	Ebonyi	30	23rd	Oyo	20	31st	Ogun	16
4th	Gombe	73	14th	Kano	29	24th	Ondo	19	34th	Anambra	14
5th	Jigawa	69	15th	Niger	28	24th	Osun	19	35th	Edo	13
6th	Taraba	67	15th	Kogi	28	26th	Federal Capital Territory	18	35th	Delta	13
7th	Bauchi	60	17th	Bayelsa	27	27th	Ekiti	18	37th	Lagos	10
8th	Sokoto	57	17th	Plateau	27	28th	Enugu	17			
9th	Borno	56	19th	Akwa Ibom	25	28th	Kwara	17			
10th	Katsina	47	20th	Nasarawa	24	30th	Imo	16			

A geopolitical zone‐based descriptive analysis of the COVID‐19 SVIs showed that the states in the northern zones of Nigeria had the highest mean SVIs. The zone with the widest range of SVI was Northwestern Nigeria (Table 2).

**TABLE 2:** Descriptive statistics on search volume index (SVI) per geopolitical zone of Nigeria.

Geopolitical Zone	TNS	NS	SVI			Rank^a^
			Range	Mean	Mode	
Southwest	6	Ekiti, Lagos, Ogun, Ondo, Osun, Oyo	10–20	17	19	4th
Southeast	5	Abia, Anambra, Ebonyi, Enugu, Imo	14–30	18.6	16	5th
South–south	6	Akwa Ibom, Bayelsa, Cross River, Rivers, Edo, and Delta	13–27	19.5	13	6th
North central	7	Benue, Kogi, Kwara, Nasarawa, Niger, Plateau, and Federal Capital Territory	17–44	26.6	28	3rd
Northeast	6	Adamawa, Bauchi, Borno, Gombe, Taraba, Yobe	37–93	64.3	Nil	1st
Northwest	7	Jigawa, Kaduna, Kano, Katsina, Kebbi, Sokoto and Zamfara	23–100	58.6	Nil	2nd

Abbreviations: NS, names of states; TNS, total number of states.

^a^ Ranking was based on mean SVI.

The lists of the top 10 related topics and queries on COVID‐19 are depicted in Table 3. “Coronavirus disease 2019” (SVI = 100), “loan” (SVI = 23), and “vaccine” or “COVID‐19 vaccine” (SVI = 6) were the top three related topics, whereas “COVID‐19 loan” (SVI = 100), “COVID‐19 Nigeria” (SVI = 78) and “COVID‐19 in Nigeria” (SVI = 58) were the top three related queries.

**TABLE 3:** The list of top 10 related topics and queries on COVID‐19.

Top related topics			Top related queries		
Rank	Topic	SVI	Rank	Query	SVI
1st	Coronavirus disease 2019	100	1st	COVID‐19 loan	100
2nd	Loan	23	2nd	COVID‐19 Nigeria	78
3rd	Vaccine	6	3rd	COVID‐19 in Nigeria	58
3rd	COVID‐19 vaccine	6	4th	COVID	54
5th	Coronavirus	4	5th	COVID‐19	39
5th	Funding	4	6th	COVID‐19 vaccine	22
7th	Symptoms of COVID‐19	3	7th	COVID‐19 news	21
7th	Preventive healthcare	3	8th	NIRSAL	19
7th	Central Bank of Nigeria	3	9th	NIRSAL COVID‐19 loan	18
10th	Pandemic	2	9th	NIRSAL loan	18

Abbreviation: SVI, search volume index.

The lists of the top 10 rising related topics and queries on COVID‐19 are depicted in Table 4. All these 10 topics and queries had a percentage increase in search by >5000%. However, “Coronavirus disease 2019,” “loan” and “vaccine” were the top three rising related topics, whereas “COVID‐19 loan,” “COVID‐19 Nigeria” and “COVID‐19 in Nigeria” were the top three rising associated queries.

**TABLE 4:** The list of top 10 rising related topics and queries on COVID‐19.

Rising related topics			Rising related queries		
Rank	Topic	PI	Rank	Query	PI
1st	Coronavirus disease 2019	Breakout	1st	COVID‐19 loan	Breakout
2nd	Loan	Breakout	2nd	COVID‐19 Nigeria	Breakout
3rd	Vaccine	Breakout	3rd	COVID‐19 in Nigeria	Breakout
4th	COVID‐19 vaccine	Breakout	4th	COVID	Breakout
5th	Coronavirus	Breakout	5th	COVID‐19	Breakout
6th	Funding	Breakout	6th	COVID‐19 vaccine	Breakout
7th	Symptoms of COVID‐19	Breakout	7th	COVID‐19 news	Breakout
8th	Preventive healthcare	Breakout	8th	NIRSAL	Breakout
9th	Central Bank of Nigeria	Breakout	9th	NIRSAL COVID‐19 loan	Breakout
10th	Pandemic	Breakout	10th	NIRSAL loan	Breakout

Abbreviation: PI, percentage increase.

### Research interest

**FIGURE 2:**
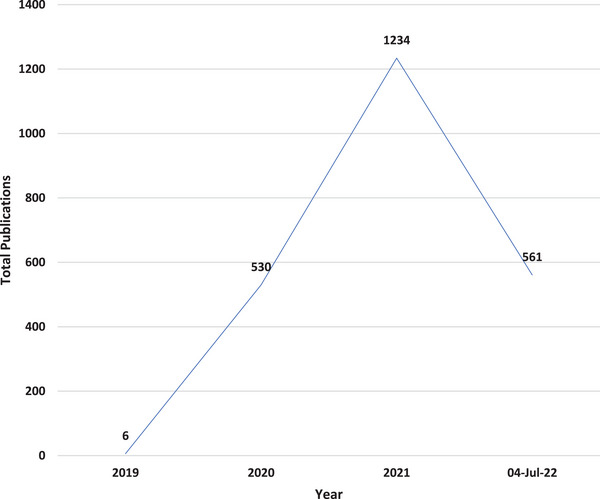
Trend analysis of annual outputs on COVID‐19 on the SCOPUS database (2019 to 04 July 2022).

A total of 2331 publications on COVID‐19, published from 2019 till 04 July 2022 are available on the SCOPUS database (Figure 2). The year‐trend of the volume of these outputs steadily increased from 2019 to 2021. Between January 2022 and 04 July 2022, only 561 outputs were published, and this was less than half of the volume of what was published in the year 2021.

**FIGURE 3:**
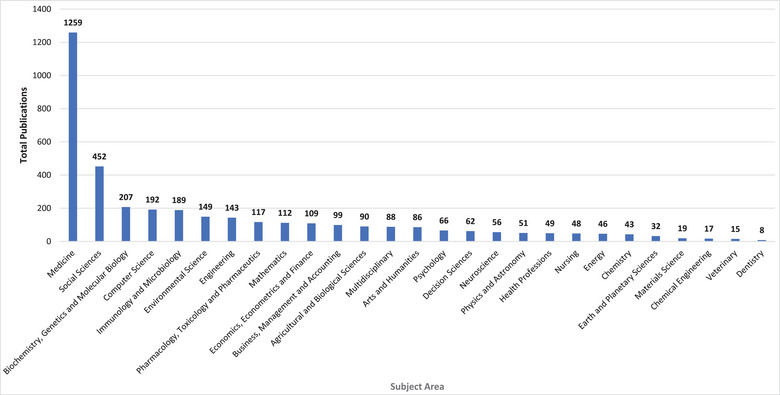
The distribution of COVID‐19 publications in Nigeria by subject areas.

The distribution of these COVID‐19 publications is depicted in Figure 3. The top three subject areas with the greatest volume of these publications were Medicine (TP = 1259), Social Sciences (TP = 452) and Biochemistry, Genetics and Molecular Biology (TP = 252), whereas the three subject areas with the smallest volume of these publications were Dentistry (TP = 8), Veterinary (TP = 15) and Chemical Engineering (TP = 17).

The distribution of COVID‐19 publications in Nigeria by publication type is depicted in Table 5. The top three most common publication types were article (TP = 1555 [66.7%]; *h*‐index = 44), review (TP = 295 [12.7%]; *h*‐index = 27) and letter (TP = 146 [6.3%]; *h*‐index = 16).

**TABLE 5:** Distribution of COVID‐19 publications in Nigeria by publication type.

Publication type	TP (%)	TC	ACP	*h*‐Index	Rank^a^
Article	1555 (66.7)	10017	6.4	44	1st
Review	295 (12.7)	4002	13.6	27	2nd
Letter	146 (6.3)	732	5.0	16	3rd
Note	115 (4.9)	1809	15.7	19	4th
Conference paper	82 (3.5)	70	0.9	5	5th
Book chapter	75 (3.2)	87	1.2	6	6th
Editorial	37 (1.6)	335	9.1	8	7th
Data paper	10 (0.4)	128	12.8	5	8th
Erratum	9 (0.4)	3	0.3	1	9th
Short survey	7 (0.3)	70	10	3	10th

Abbreviations: ACP, average citations per paper; TC, total citations; TP, total publications.

^a^ Rank was based on TP.

**FIGURE 4:**
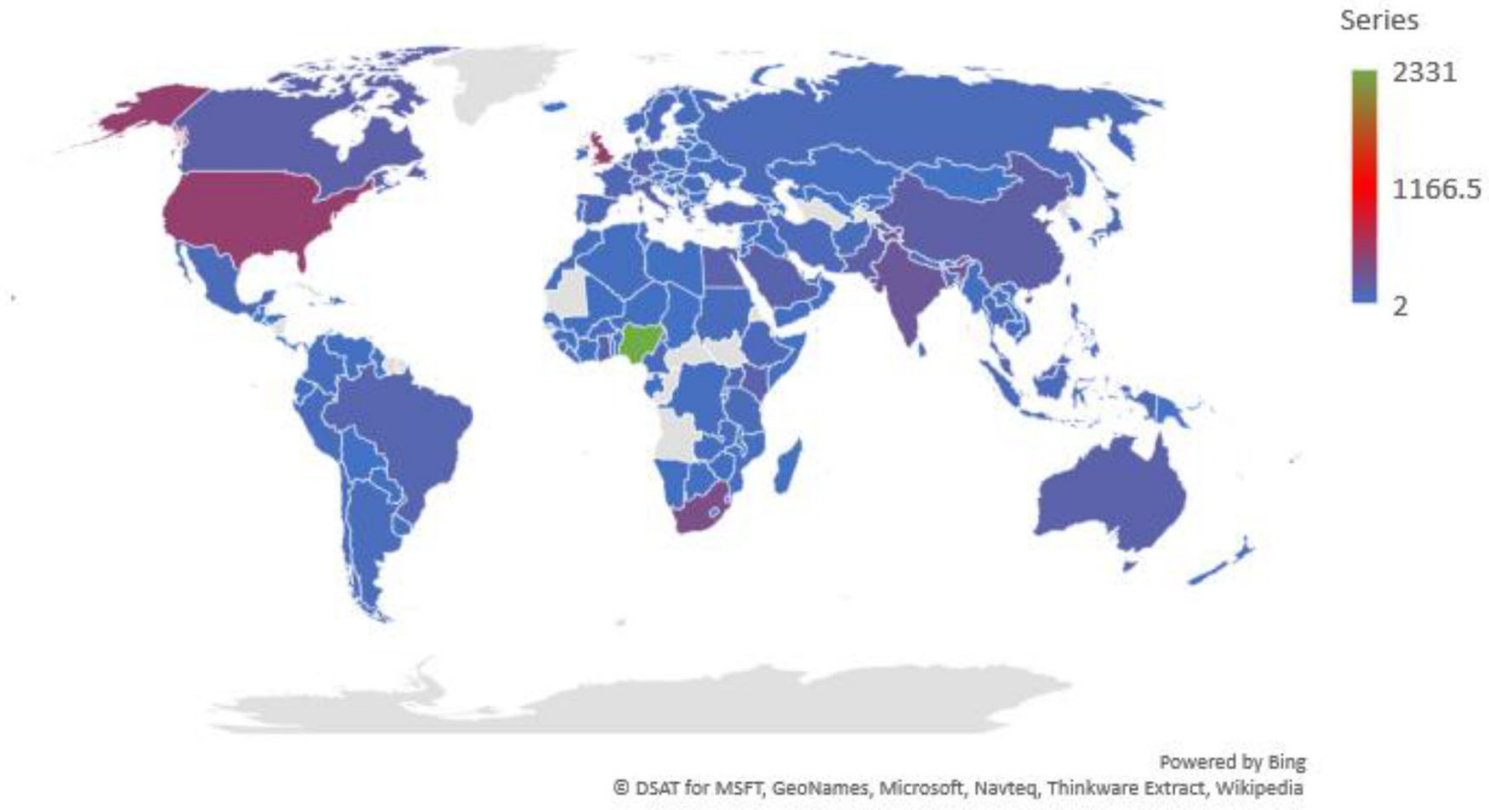
Global distribution of countries/territories/dependencies that have collaborated with Nigeria in COVID‐19 publications.

A total of 159 countries/territories/dependencies had collaborated with Nigeria in COVID‐19 publications (Figure 4). However, the top 10 countries collaborating with Nigeria with the highest volume of productivity were the United Kingdom (TP = 509 [21.8%]; *h*‐index = 38), USA (TP = 502 [21.5%]; *h*‐index = 35), and South Africa (TP = 348 [14.9%]; *h*‐index = 26). Pertinently, only South Africa and Egypt were the only African countries/territories/dependencies that made this list (Table 6).

**TABLE 6:** List of top 10 countries collaborating with Nigeria in COVID‐19 research.

Country	Continent	TP (%)	TC	ACP	*h*‐Index	Rank^a^
United Kingdom	Europe	509 (21.8)	7736	15.2	38	1st
United States	North America	502 (21.5)	6988	13.9	35	2nd
South Africa	Africa	348 (14.9)	2676	7.7	26	3rd
India	Asia	259 (11.1)	4261	16.5	27	4th
China	Asia	173 (7.4)	1809	10.5	21	5th
Pakistan	Asia	165 (7.1)	2117	12.8	22	6th
Canada	North America	163 (7.0)	4256	26.1	25	7th
Australia	Australia	152 (6.5)	3176	20.9	22	8th
Egypt	Africa	144 (6.2)	1594	11.1	21	9th
Saudi Arabia	Asia	140 (6.0)	2187	15.6	22	10th

Abbreviations: ACP, average citations per paper; TC, total citations; TP, total publications.

^a^ Rank was based on TP.

Table 7 shows the list of the top 10 local institutions in Nigeria sourcing COVID‐19 publications. All those institutions that made the list were owned by the Federal Government of Nigeria, and seven tenths of them were situated in the southern parts of Nigeria – majority (71.4% [5/7]) of which were in the Southwestern Nigeria geopolitical zone. The three most productive institutions were University of Ibadan (*n* = 368 [15.8%]; *h*‐index = 27), University of Nigeria (TP = 226 [9.7%]; *h*‐index = 13) and University of Ilorin (TP = 153 [6.6%]; *h*‐index = 21).

**TABLE 7:** The list of the top 10 local institutions in Nigeria sourcing COVID‐19 publications.

Local institutions	GPZ	Ownership	TP (%)	TC	ACP	*h*‐Index	Rank^a^
University of Ibadan	SWN	FG	368 (15.8)	3204	8.7	27	1st
University of Nigeria	SEN	FG	226 (9.7)	800	3.5	13	2nd
University of Ilorin	NCN	FG	153 (6.6)	1426	9.3	21	3rd
University of Lagos	SWN	FG	142 (6.1)	1183	8.3	16	4th
Bayero University	NWN	FG	110 (4.7)	1122	10.2	19	5th
Ahmadu Bello University	NWN	FG	106 (4.5)	612	5.8	14	6th
Obafemi Awolowo University	SWN	FG	99 (4.2)	565	5.7	13	7th
University College Hospital	SWN	FG	96 (4.1)	2126	22.1	15	8th
Federal University of Technology Akure	SWN	FG	90 (3.9)	829	9.2	14	9th
University of Calabar	SSN	FG	87 (3.8)	778	8.9	12	10th

Abbreviations: ACP, average citations per paper; FG, Federal Government; GPZ, geopolitical zone; NCN, North Central Nigeria; NWN, Northwestern Nigeria; SEN, Southeastern Nigeria; SSN, Southsouthern Nigeria; SWN, Southwestern Nigeria; TC, total citations; TP, total publications.

^a^ Rank was based on TP.

Table 8 shows the list of the top 10 foreign institutions collaborating with Nigeria in sourcing COVID‐19 publications. All those institutions that made the list were owned by governments, and none was privately owned. Only four tenths of these institutions were situated in a foreign African country, and all of them were in South Africa. The top three most productive foreign collaborating institutions were the London School of Hygiene and Tropical Medicine (TP = 116 [5.0%]; *h*‐index = 20), University of Kwazulu‐Natal (TP = 67 [2.9%]; *h*‐index = 13) and University of Cape Town (TP = 65 [2.8%]; *h*‐index = 11).

**TABLE 8:** The list of the top 10 foreign institutions collaborating with Nigeria in sourcing COVID‐19 publications.

Foreign institutions	Country	Ownership	TP	TC	ACP	*h*‐Index	Rank^a^
London School of Hygiene and Tropical Medicine	United Kingdom	Government	116 (5.0)	1982	17.1	20	1st
University of Kwazulu‐Natal	South Africa	Government	67 (2.9)	734	11.0	13	2nd
University of Cape Town	South Africa	Government	65 (2.8)	434	6.7	11	3rd
Organisation Mondiale de la Santé (World Health Organization)	Switzerland	Government	63 (2.7)	439	7.0	12	4th
University College of London	United Kingdom	Government	62 (2.7)	2568	41.4	17	5th
Imperial College London	United Kingdom	Government	47 (2.0)	439	9.3	13	6th
University of Pretoria	South Africa	Government	46 (2.0)	273	5.9	9	7th
Karolinska Institutet	Sweden	Government	44 (1.9)	1537	34.9	14	8th
Stellenbosch University	South Africa	Government	43 (1.8)	603	14.0	12	9th
Universiti Sains Malaysia	Malaysia	Government	40 (1.7)	672	16.8	13	10th

Abbreviations: ACP, average citations per paper; TC, total citations; TP, total publications.

^a^ Rank was based on TP.

Table 9 shows the list of top 10 Nigerian authors of COVID‐19 publications. The majority (60% [6/10]) of these authors were affiliated to institutions situated in the Southwestern Nigeria geopolitical zone. Adebisi YA (TP = 47 [2.0%]; *h*‐index = 11), affiliated to the University of Ibadan; Aborode AT (TP = 35 [1.5%]; *h*‐index = 9), affiliated to the Healthy Africans Platform; and Ihekweazu CA (TP = 26 [1.1%]; *h*‐index = 11), affiliated to the Nigeria Centre for Disease Control were the top three authors in this list.

**TABLE 9:** The list of top 10 Nigerian authors of COVID‐19 publications.

Authors	Institution	GPZ	TP (%)	TC	ACP	*h*‐Index	Rank^a^
Adebisi, Yusuff Adebayo	University of Ibadan	SWN	47 (2.0)	347	7.4	11	1st
Aborode, Abdullahi Tunde	Healthy Africans Platform	SWN	35 (1.5)	175	5.0	9	2nd
Ihekweazu, Chikwe A	Nigeria Centre for Disease Control	NCN	26 (1.1)	1162	44.7	11	3rd
Ilesanmi, Olayinka Stephen	University of Ibadan	SWN	24 (1.0)	152	6.3	7	4th
Salisu, Afees Adebare	University of Ibadan	SWN	22 (0.9)	540	24.5	9	5th
Folayan, Morenike O	Obafemi Awolowo University	SWN	21 (0.9)	198	9.4	7	6th
Sam‐Agudu, Nadia Adjoa	Institute of Human Virology	NCN	20 (0.9)	363	18.2	9	7th
Musa, Salihu Sabiu	Kano University of Science and Technology	NWN	19 (0.8)	154	8.1	7	8th
Awotunde, Joseph Bamidele	University of Ilorin	NCN	18 (0.7)	79	4.4	5	9th
Adiukwu, Frances N	University of Port Harcourt	SSN	15 (0.6)	284	18.9	7	10th

Abbreviations: ACP, average citations per paper; FG, Federal Government; GPZ, geopolitical zone; NCN, North Central Nigeria; NWN, Northwestern Nigeria; SEN, Southeastern Nigeria; SSN, Southsouthern Nigeria; SWN, Southwestern Nigeria; TC, total citations; TP, total publications.

^a^ Rank was based on TP.

Table 10 shows the list of the top 10 institutions funding COVID‐19 research projects in Nigeria. None of these institutions was headquartered in Africa; moreover, none of them was indigenous. Only 2 out of these 10 institutions were private. Those funding institutions headquartered in the USA constituted the majority (50% [5/10]) of those institutions in this list. The top three institutions in the list were the National Institutes of Health (TP = 72 [3.1%]; *h*‐index = 14), Bill and Melinda Gates Foundation (TP = 36 [1.5%]; *h*‐index = 12) and Wellcome Trust (TP = 32 [1.4%]; *h*‐index = 9).

**TABLE 10:** The list of the top 10 institutions funding COVID‐19 research projects in Nigeria.

Funding Institutions	Ownership	HQ (country)	TP (%)	TC	ACP	*h*‐Index	Rank^a^
National Institutes of Health	Government	USA	72 (3.1)	742	10.3	14	1st
Bill and Melinda Gates Foundation	Private	USA	36 (1.5)	575	16.0	12	2nd
Wellcome Trust	Private	United Kingdom	32 (1.4)	1548	48.4	9	3rd
US Department of Health and Human Services	Government	USA	26 (1.1)	133	5.1	6	4th
Fogarty International Center	Government	USA	24 (1.0)	406	16.9	10	5th
National Institute for Health Research	Government	United Kingdom	23 (1.0)	339	14.7	7	6th
World Health Organization	Government	Switzerland	21 (0.9)	153	7.3	7	7th
European Commission	Government	Belgium	20 (0.9)	183	9.2	8	8th
National Institute of Allergy and Infectious Diseases	Government	USA	20 (0.9)	1101	55.1	10	9th
Medical Research Council	Government	United Kingdom	18 (0.8)	1448	80.4	7	10th
							

Abbreviations: ACP, Average citations per paper; HQ, Headquarters; TC, Total citations; TP, Total publications.

^a^ Rank was based on TP.

Table 11 shows the list of the top 10 journals publishing COVID‐19 papers authored by Nigerian researchers. Only one of the publishers of these journals was headquartered in Kenya, whereas the rest were in the USA, United Kingdom, the Netherlands and Switzerland. Amongst these journals, the Journal of Medical Virology had the highest CiteScore 2021 (*n* = 18.8). The top three most productive journals were Pan African Medical Journal (TP = 118 [5.1%]; *h*‐index = 9), American Journal of Tropical Medicine and Hygiene (TP = 37 [1.6%]; *h*‐index = 12) and PLoS One (TP = 34 [1.5%]; *h*‐index = 7).

**TABLE 11:** The list of the top 10 journals publishing COVID‐19 papers authored by Nigerian researchers.

Journals	CiteScore 2021	Publisher (HQ)	TP (%)	TC	ACP	*h*‐Index	Rank^a^
Pan African Medical Journal	1.0	Pan African Medical Journal (Kenya)	118 (5.1)	382	3.2	9	1st
American Journal of Tropical Medicine and Hygiene	4.4	American Society of Tropical Medicine and Hygiene (USA)	37 (1.6)	395	10.7	12	2nd
PLoS One	5.6	Public Library of Science (USA)	34 (1.5)	235	6.9	7	3rd
BMJ Global Health	7.2	BMJ Publishing Group (United Kingdom)	30 (1.3)	475	15.8	9	4th
Library Philosophy and Practice	0.4	University of Nebraska‐Lincoln (USA)	29 (1.2)	25	0.9	3	5th
International Journal of Infectious Diseases	10.8	Elsevier (the Netherlands)	21 (0.9)	477	22.7	11	6th
Journal of Global Health	5.1	Edinburgh University Global Health Society (United Kingdom)	20 (0.9)	62	3.1	3	7th
Journal of Medical Virology	18.8	Wiley‐Blackwell (USA)	17 (0.7)	234	13.8	9	8th
Scientific African	2.4	Elsevier (the Netherlands)	17 (0.7)	38	2.2	2	8th
Frontiers in Public Health	4.0	Frontiers Media S.A. (Switzerland)	16 (0.7)	56	3.5	5	10th

Abbreviations: ACP, Average citations per paper; HQ, Headquarters; TC, Total citations; TP, Total publications.

^a^ Rank was based on TP.

## DISCUSSION

COVID‐19 pandemic, as it is novel, is characterised by the need for information. This study is an infoveillance and bibliometric research to obtain COVID‐19 information prevalence and COVID‐19 research productivity in Nigeria. This study aims not only to examine the spread of accurate and inaccurate information but also to assess the information‐wish and efforts in getting information by the Nigerian population, trending nuances and research incidence, among others. The information search spike started 1 week before the first confirmed case. The search spike conforms with a previous analysis that online searches follow COVID‐19 trend, with early online activities (mostly 12 days) before confirmed cases and deaths [16, 17]. The early search spike in Nigeria coincided with the information from the Minister of Health that the country was prepared for a possible COVID‐19 outbreak.

The Minister was quoted to have said that Nigeria was “prepared and ready to contain the novel coronavirus disease, if eventually, it breaks out in Nigeria” [18]. The Minister made the statement when inspecting facilities at the Emergency Response Readiness for COVID‐19 victims in the country entry hub, Lagos. At that time, the Minister could not have estimated the possible magnitude of the pandemic but believed that the country's robust surveillance system and lessons learned from the Ebola episode of 2014 would help tackle the impeding COVID‐19 [18]. There were a lot of reactions and counter‐reaction, and information wishes. This could explain the spike in online information searches across Nigeria about COVID‐19.

SVI inequalities were observed across the country, with the northern parts of Nigeria having a higher search volume for COVID‐19. Northern Nigeria generally has a lower internet penetration and search density than the South. Hence, COVID‐19 dominated the search item within the period. The 21st century has been characterised by the effective use of internet searches, mostly by the youth. The internet is an established medium for health information and a primary socialising agent concerning health and illness behaviour. Despite the challenges of online health information search, such as unreliable and slow connection, high cost of Internet and unreliable power supply in most low‐income countries, most people still rely on online health information for lay diagnosis and general health support [19, 20, 21].

This study also uncovered several top search terms, including “COVID‐19,” “COVID loan” and “vaccine,” and queries, including “COVID‐19 Nigeria,” “COVID loan” and “COVID‐19 in Nigeria,” among others. This shows critical epidemiologic concerns in Nigeria. Previous studies show that disease symptoms are top search priorities [16, 22]. This study indicates that Nigerians’ concern for livelihood survival strategies, such as COVID‐19 loans, supersedes the symptom concern. These search terms and queries are important in understanding infodemiology vis à vis public interests/concerns. The first search concern is general information about the disease followed, much later, by personal health strategies [22].

This study shows the need to understand the imperative of online health information‐seeking behaviour and online health communication, which has been a significant crux in COVID‐19 infodemiology or infoveillance, especially during the pandemic (see also [23, 24]). Perhaps, it was not surprising that a higher incidence of COVID‐19 cases was translated to a higher number of COVID‐19 queries on Google [25]. The Internet became a truth or fact‐seeking platform because of the available volume of unsolicited online COVID‐19 information and hashtags, especially on social media. Hence, the monikers “coronavirus 5G,” “coronavirus conspiracy” and “tips and cures for COVID‐19” trended during the pandemic [25]. Understanding the infodemic patterns and search terms will influence mass media regulators and health authorities to be vigilant and tackle the spread of misinformation.

This study also noted the spike in or emerged research interest of Nigerian researchers concerning COVID‐19. The research interest was not compared to other countries. However, the pandemic trend positively correlated with the research outputs or productivity concerning COVID‐19 in most countries, including Nigeria (see also [26, 27, 28]). There was also a research output spike during the pandemic, and as the global prevalence increased, so was the number of scientific reports/articles. The emerged COVID‐19 research interest is without prejudice to the general disruption of the scientific enterprise [29], mainly due to lockdown and deglobalisation. For instance, a decline in hours spent on research and in new research projects and co‐authorship has been reported [29]. It is thus essential to acknowledge the scientific resilience during the pandemic, which generated commendable research outputs in Nigeria and elsewhere. The research trend in Nigeria stably confirms the assertion that research productivity during the COVID‐19 pandemic “correlates with epidemiologic, health care system‐related and health economic factors, and pre‐COVID publication expertise” [26]. Hence, most countries with research culture maintained scientific resilience to produce COVID‐19 publications at high rates [26].

The research productivity cuts across various disciplines. The top three subject areas with the most significant volume of these publications were Medicine, Social Sciences and Biochemistry, Genetics and Molecular Biology. A multidisciplinary approach, to COVID‐19 research, with the involvement of social sciences, is imperative, and critical for future pandemic prevention and preparedness (see also [30, 31, 32]). There was also magnificent collaboration for COVID‐19 research. This study found that researchers from over 150 countries collaborated with Nigerian researchers on COVID‐19 research. However, the top 10 collaborating countries with the highest volume of productivity were the United Kingdom, USA and South Africa. This research result contradicted a submission that collaborations worldwide were strongly related to geographical location [33]. The top three most productive foreign collaborating institutions were the London School of Hygiene and Tropical Medicine, University of Kwazulu‐Natal and University of Cape Town. However, none of the top 10 institutions funding COVID‐19 research projects in Nigeria is a local institution or headquartered in Africa. The USA funding institutions constituted the majority (50% [5/10]) of those institutions in the top 10. This further shows that most research funding in Nigeria and Africa comes from external funding (see also [34, 35]), but Africa's future research agenda should greatly be shaped by local funding and support.

## CONCLUSION

In almost all countries, internet searches for COVID‐related information were massive, with significant effects on control efforts. It signifies the need for information, a kind of void which the concerned authorities should fill. Information consumption must be controlled, especially during emergencies, to ensure that accurate information overflows the inaccurate ones. This indicates that online search data can be used to develop public health infoveillance intervention which will help inform future emergency response. The utility of digital information epidemiology in providing valuable surveillance data of disease outbreaks should always be a priority. Internet search spikes reflect clinical manifestations of the disease and public concerns, which often correlate significantly with real‐world cases and deaths. There was scientific resilience in the form of COVID‐19 research output during the pandemic. The research productivity cuts across various disciplines with magnificent collaboration across various countries. This research resilience depicts great potential, hence, a call for improved local funding for research and development.

## AUTHOR CONTRIBUTIONS

*Conceptualisation; resources; supervision; validation; writing – original draft; writing – review and editing*: Jimoh Amzat. *Conceptualisation; data curation; formal analysis; funding acquisition; investigation; methodology; project administration; resources; software; supervision; validation; visualisation; writing – original draft; writing – review and editing*: Kehinde Kazeem Kanmodi. *Data curation; formal analysis; resources*: Eyinade Adeduntan Egbedina. Jimoh Amzat and Kehinde Kazeem Kanmodi contributed to this paper equally.

## CONFLICT OF INTEREST STATEMENT

Authors have no conflict of interest to declare.

## FUNDING INFORMATION

This study was self‐funded.

## ETHICS STATEMENT

Not applicable.

## Data Availability

Data sharing is not applicable to this article as no new data were created or analysed in this study.
